# Exercise‐induced alterations in pancreatic oxidative stress and mitochondrial function in type 2 diabetic Goto‐Kakizaki rats

**DOI:** 10.14814/phy2.12751

**Published:** 2016-04-19

**Authors:** Haider Raza, Annie John, Jasmin Shafarin, Frank C. Howarth

**Affiliations:** ^1^ Department of BiochemistryCollege of Medicine and Health Sciences, United Arab Emirates UniversityAl AinUnited Arab Emirates; ^2^ Department of PhysiologyCollege of Medicine and Health Sciences, United Arab Emirates UniversityAl AinUnited Arab Emirates

**Keywords:** Energy metabolism, exercise, GK rats, mitochondria, oxidative stress, Pancreas, type 2 diabetes

## Abstract

Progressive metabolic complications accompanied by oxidative stress are the hallmarks of type 2 diabetes. The precise molecular mechanisms of the disease complications, however, remain elusive. Exercise‐induced nontherapeutic management of type 2 diabetes is the first line of choice to control hyperglycemia and diabetes associated complications. In this study, using 11‐month‐old type 2 Goto‐Kakizaki (GK) rats, we have investigated the effects of exercise on mitochondrial metabolic and oxidative stress in the pancreas. Our results showed an increase in the NADPH oxidase enzyme activity and reactive oxygen species (ROS) production in GK rats, which was inhibited after exercise. Increased lipid peroxidation and protein carbonylation and SOD activity were also inhibited after exercise. Interestingly, glutathione (GSH) level was markedly high in the pancreas of GK diabetic rats even after exercise. However, GSH‐peroxidase and GSH‐reductase activities were significantly reduced. Exercise also induced energy metabolism as observed by increased hexokinase and glutamate dehydrogenase activities. A significant decrease in the activities of mitochondrial Complexes II/III and IV were observed in the GK rats. Exercise improved only Complex IV activity suggesting increased utilization of oxygen. We also observed increased activities of cytochrome P450s in the pancreas of GK rats which was reduced significantly after exercise. SDS‐PAGE results have shown a decreased expression of NF‐*κ*B, Glut‐2, and PPAR‐ϒ in GK rats which was markedly increased after exercise. These results suggest differential oxidative stress and antioxidant defense responses after exercise. Our results also suggest improved mitochondrial function and energy utilization in the pancreas of exercising GK rats.

## Introduction

Prevalence of type 2 diabetes, the most common metabolic disorder, is increasing worldwide and in the Middle East in particular (Majeed et al. 2014). In 2013, 382 million people had diabetes; this number is expected to increase to 592 million by 2035 (Guariguata et al. 2014). Type 2 diabetes is accompanied by hyperglycemia, hyperlipidemia, increased inflammation and oxidative stress, and other energy‐related metabolic complications, characterized by impaired insulin response, insulin resistance, or both. Goto‐Kakizaki (GK) nonobese rats are genetic models of type 2 diabetes developed by in‐breeding Wistar rats with a selection of rats in each generation with the highest blood glucose levels estimated by glucose tolerance test (Ostenson et al. 1993). Type 2 diabetic rats exhibit progressive alterations in energy metabolism (Boudina and Abel 2007; Golbidi and Laher 2011). Type 2 diabetic rats with metabolic syndrome also display a cluster of different metabolic abnormalities including insulin resistance, hyperglycemia, hypertriglyceridemia, increased inflammatory biomarkers, and altered drug metabolism (Chander et al. 2004). The precise molecular mechanism of progressive metabolic complications and insulin resistance in GK diabetic rats is not completely understood.

Our previous studies in GK rats fed a high fat diet or subjected to exercise training have shown differential cardiovascular responses and alterations in calcium signaling, progressive hyperglycemia, and metabolic complications in comparison to the Wistar control rats (Howarth et al. 2011; Salem et al. 2013). Studies have suggested that prolonged hyperinsulinemia in type 2 diabetes, suppresses mitochondrial biogenesis and function and this may lead to impairment of insulin sensitivity (Zierath 2002; Liu et al. 2009). Our recent studies in Zucker Diabetic Fatty (ZDF) rats, another commonly used animal model of type 2 diabetes, have shown increased oxidative stress and mitochondrial dysfunction (Raza et al. 2012, 2013, 2015). Our previous studies in STZ‐induced type 1 diabetic rat model have also demonstrated alterations in mitochondrial functions and oxidative‐stress‐related complications in different tissues (Raza et al. 2004, 2011). These studies have demonstrated that increased oxidative stress due to increased production of reactive oxygen species (ROS), lipid/protein peroxidation, and nitric oxide (NO) production, particularly in the pancreas, have resulted in alterations in glutathione (GSH)‐dependent antioxidant defense metabolism which protects cells from diabetes‐associated complications.

Alterations in metabolic homeostasis, energy usage, oxidative phosphorylation, and ROS production are dependent on mitochondrial functions. Exercise is considered to be one of the most valuable nonpharmacological approaches in the management of type 2 diabetes. However, exercise may perturb metabolic homeostasis and enhance the utilization of nutrients and oxygen which may alter mitochondrial functions. The precise mechanism of improved metabolic functions after exercise training and its effects on redox homeostasis is not clear. Mitochondrial function is the key to metabolic homeostasis in metabolizing nutrients into ATP and also in generating ROS which act as secondary messengers to mediate metabolism at the lower level but is detrimental to mitochondrial function at higher concentrations (Cheng and Ristow 2013). Both clinical and experimental studies (Higuchi et al. 1985; Hafstad et al. 2013, 2015; Sacre et al. 2014; Gomez‐Cabrera et al. 2015) have shown that exercise can induce cardioprotection in normal hearts as well as in diabetes and obesity and therefore is recommended for nontherapeutic management of type 2 diabetes. Studies on type 2 diabetic rats have also shown improved glucose tolerance (Diabetes Prevention Program Research Group, 2002) and glucose‐dependent insulin release after exercise. Loss of *β*‐cell structure and function is seen in diabetes. Exercise improves islet architecture, *β*‐cell viability, and insulin content (Rawal et al. 2013; Wang et al. 2013; Paula et al. 2015). Moderate‐intensity exercise training appears to improve diabetic status, insulin content, and glucose regulation better than low‐ or high‐intensity exercise training (Traisaeng et al. 2014). Intensity of exercise (low, moderate, and high) can therefore differently affect the delicate balance between glucose utilization and glucose production as well as insulin secretion in diabetes. However, the effects of exercise on mitochondrial, metabolic, oxidative, and redox functions in pancreas are still not clear. Therefore, we have studied the effects of exercise on metabolic and redox homeostasis in the pancreas of GK diabetic rats and compared them with nondiabetic Wistar rats. In addition, we have studied the effects of moderate exercise training on metabolic adaptation in these rats.

## Materials and Methods

### Chemicals and reagents

Cytochrome c, reduced glutathione (GSH), oxidized glutathione (GSSG), 5,5′‐dithio‐bis(2‐nitrobenzoic acid), cumene hydroperoxide, dimethylnitrosamine (DMNA), erythromycin, 7‐ethoxyresorufin, methoxyresorufin, resorufin, dinitrophenylhydrazine (DNPH), lucigenin, glutathione reductase, thiobarbituric acid, NADH, NADPH, coenzyme Q2, sodium succinate, antimycin A, rotenone, dodecyl maltoside, ATP Bioluminescent cell assay kits, and Hexokinase assay kits were purchased from Sigma‐Aldrich Fine Chemicals (St Louis, MO). 2′, 7′‐Dichlorofluorescein diacetate (DCFDA) was procured from Molecular Probes (Eugene, OR). Kits for SOD assay were procured from R & D Systems (Minneapolis, MN) and for glutamate dehydrogenase assay from Abcam (Cambridge, UK).Polyclonal antibodies against CYP2E1, Glut 2, NF‐kB p65, and *β*‐actin were purchased from Santa Cruz Biotechnology Inc. (Santa Cruz, CA), whereas those against PPAR‐*γ* and HO‐1 were purchased from Abcam. Reagents for SDS‐PAGE and Western blot analyses were purchased from Gibco BRL (Grand Island, NY) and Bio Rad Laboratories (Richmond, CA).

### Animals and exercise regimen

Thirty male GK (Taconic, Germantown, NY) and 30 male Wistar control rats aged 11 months were divided into four subgroups, each containing 15 animals. Two subgroups of GK and control rats received exercise training, whereas the other two subgroups of GK and control rats continued a sedentary life style. Exercise training was performed on a treadmill (EXER‐4; Columbus Instruments, Columbus, OH) as described before with minor modifications (Salem et al. 2013). Briefly, daily 1 h exercise training sessions were repeated 5 days per week for a period of 8 weeks. Each exercise training period began with 10 min warm‐up during which time the belt speed was gradually increased from zero to training speed. During week 1, the belt speed was 10 m/min, during weeks 2–3 belt speed was increased at 15 m/min, and during week 4 belt speed was maintained at 15 m/min and the belt gradient was increased from 0^0^ to 10^0^. Thereafter, the exercise training belt speed was increased to 20 m/min. Regular body weight, heart weight/body weight ratio, and fasting and nonfasting glucose were continuously monitored and 2 months into the exercise training program, animals were subjected to glucose tolerance test as described before (Salem et al. 2013). Ethical approval was obtained from the Animal Ethics Committee, College of Medicine and Health Sciences, United Arab Emirates University.

Animals were sacrificed by decapitation and the pancreas (*n* = 6–8) were quickly excised and stored at −80°C until further analysis. A portion of the tissues were homogenized in H‐medium mitochondrial isolation buffer (70 mmol/L sucrose, 220 mmol/L mannitol, 2.5 mmol/L HEPES, 2 mmol/L EDTA, 0.1 mmol/L phenylmethylsulfonylfluoride, pH 7.4) at 4°C. Cellular fractions were prepared by differential centrifugation and purity of the isolated fractions was confirmed as described by Raza et al. (Raza et al. 2004, 2011, 2012, 2013, 2015). Cellular fractions were suspended in the above buffer and analyzed immediately for respiratory function and oxidative stress. Protein concentration was measured using BioRad reagent as described before.

### Measurement of ROS

Production of ROS in diabetic and control rat pancreas was measured using 2, 7‐DCDF fluorescence assay and NADPH oxidase (NOX) activity was measured using the lucigenin‐enhanced chemiluminescence method using the Turner Designs TD‐20/20 luminometer (Raza et al. 2004, 2011, 2012, 2013, 2015).

### Measurement of SOD activity

SOD activity was measured as percent conversion of NBT to NBT‐diformazan according to the vendor's protocol (R & D Systems). The percent reduction in formazan formation was used as a measure of SOD activity.

### Protein and Lipid peroxidation (LPO) assays

Protein peroxidative carbonylation as a marker of increased oxidative stress was measured by DNPH conjugation method as described before (Raza et al. 2004, 2011, 2012, 2013, 2015). NADPH‐dependent‐membrane lipid peroxidation was measured as malonedialdehyde formed using the standard thiobarbituric acid method as described before (Raza et al. 2004, 2011, 2012, 2013, 2015).

### Measurement of GSH and GSH metabolism

GSH is the most important cellular antioxidant protecting tissues from oxidative insults. Alteration in GSH‐redox metabolism is the key indicator of perturbed antioxidant metabolism. GSH concentration was measured by NADPH‐dependent GSSG‐reductase catalyzed conversion of oxidized GSSG to GSH. Glutathione peroxidase (GSH‐Px) activity using cumene hydroperoxide and glutathione‐reductase activity using GSSG/NADPH as the respective substrates was measured by standard protocols as described by Raza et al. (Raza et al. 2004, 2011, 2012, 2013, 2015).

### Measurement of CYP 450‐dependent enzyme systems

Studies, including our own (Raza et al. 2004, 2012, 2013, 2015) have shown that drug metabolizing enzyme systems are affected in diabetes which might be implicated in developing oxidative stress, mitochondrial dysfunction, and associated complications in disease. Using erythromycin, dimethylnitrosamine, ethoxyresorufin, and methoxyresorufin as specific substrates for CYP 3A4, CYP2E1, and CYP1A1/1A2, respectively, we measured the catalytic activities in the pancreas as described before.

### Measurement of Hexokinase and glutamate dehydrogenase activities

Hexokinase activity was measured using the Hexokinase assay kit (Sigma‐Aldrich Fine Chemicals) as per the manufacturer's protocol. Briefly, it is a coupled enzyme assay, in which glucose is converted to glucose‐6‐phosphate, which is oxidized by glucose‐6‐phosphate dehydrogenase to form NADH, which in turn, reduces a colorless probe to form a colorimetric product which can be read at 450 nm.

Glutamate dehydrogenase activity was measured using the Glutamate dehydrogenase detection kit (Abcam) as per the vendor's protocol. Briefly, mitochondria from the pancreas were treated with a reaction buffer, consisting of glutamate as a specific substrate and the generation of NADH was measured spectrophotometrically at 450 nm.

### Measurement of activities of mitochondrial respiratory enzyme complexes and ATP content

Freshly isolated mitochondria (5 *μ*g protein) from pancreas were suspended in 1.0 mL of 20 mmol/L KPi buffer, pH 7.4, in the presence of the detergent, lauryl maltoside (0.2%). NADH‐ubiquinone oxidoreductase (Complex I), succinate‐cytochrome c reductase (Complex II + III), and cytochrome c oxidase (Complex IV) activities were measured using the substrates ubiquinone, succinate‐cytochrome c, and reduced cytochrome c, respectively, by the methods of Birch‐Machin and Turnbull (Birch‐Machin and Turnbull 2001) as described before (Raza et al. 2004, 2011, 2012, 2013, 2015). ATP content was also measured using the bioluminescent assay kit (Sigma, St. Louis, MO) as per the manufacturer's protocol and samples were read using the TD‐20/20 luminometer.

### SDS‐PAGE and western blot analysis

Cellular proteins (50–100 *μ*g) were electrophoretically separated by 12% SDS‐PAGE (Laemmli 1970) and transferred on to nitrocellulose paper (Towbin et al. 1979). The expression of specific enzymes and proteins was checked by immunoreactions with their specific antibodies (NF‐kB, HO‐1, CYP2E1, Glut‐2, PPAR‐*γ*) by Western blot analysis as described before (Raza et al. 2004, 2011, 2012, 2013, 2015).

### Statistical analysis

Values were calculated as mean + SEM of at least three determinations. Statistical significance of the data was assessed using SPSS software (IBM Corporation, Armonk, NY) by analysis of variance followed by Dunnett post hoc analysis, and *P* < 0.05 were considered statistically significant.

## Results

### Effect of exercise on oxidative stress in GK rat pancreas

**Figure 1:**
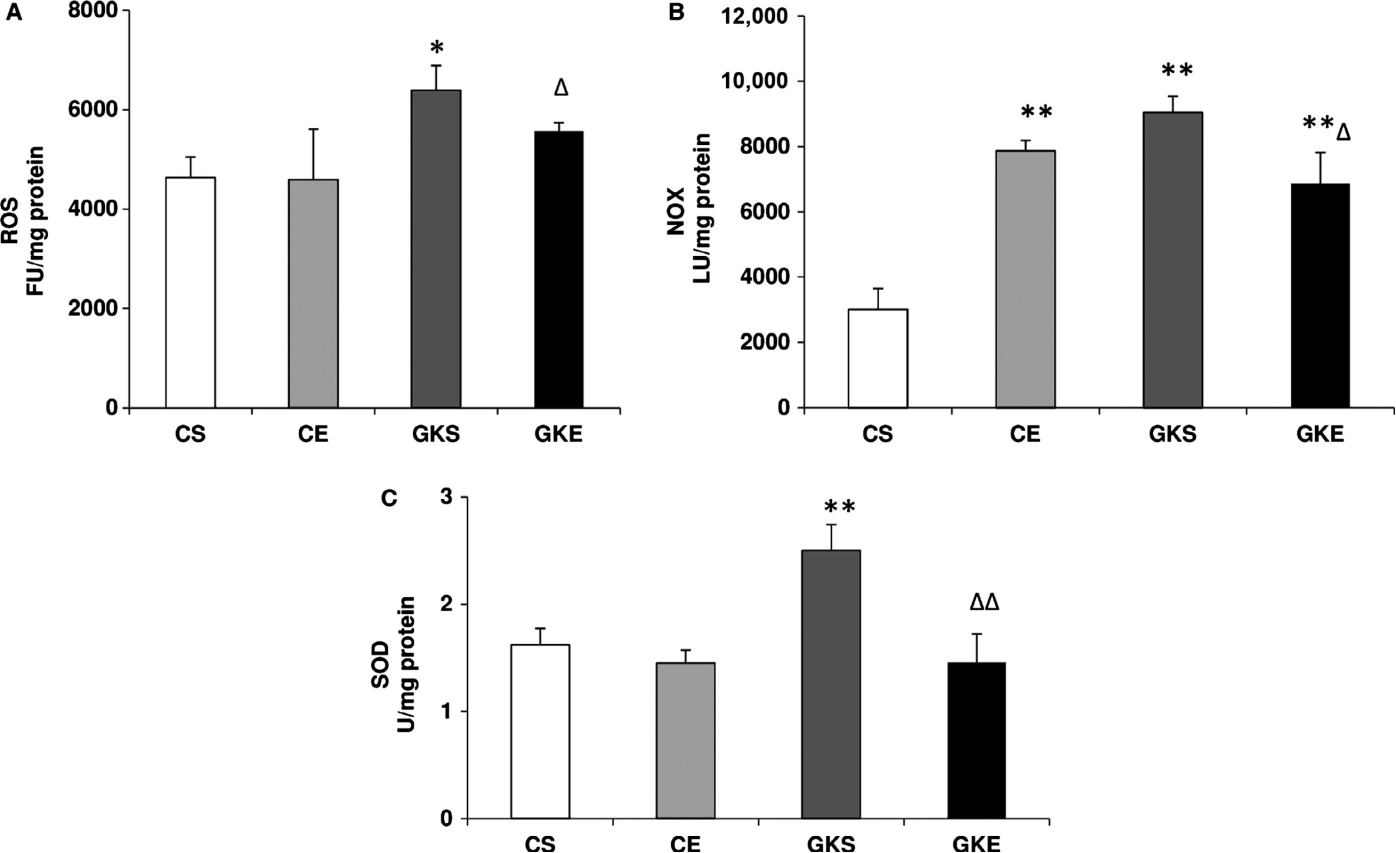
Effect of exercise on reactive oxygen species (ROS) production and SOD: The pancreas was homogenized and cellular fractions were prepared from control and Goto‐Kakizaki (GK) sedentary and exercise‐trained rats as described in the Materials and Methods. ROS (A) was measured by Dichlorofluorescein diacetate ‐ROS fluorescence using 2′, 7′‐DCDF fluorescence assay and NADPH oxidase (NOX) activity (B) was measured using the lucigenin‐enhanced chemiluminescence method using the Turner Designs TD‐20/20 luminometer as described before. SOD (C) was measured as percent conversion of NBT to NBT‐diformazan according to the vendor's protocol. The results shown are +/− SEM of three independent experiments. Asterisks indicate significant difference (*P < 0.05; **P < 0.01 from control and ∆ *P* < 0.05; ∆∆ *P* < 0.01 from GK sedentary data).

Figure 1A shows a marked increase in ROS production in the pancreas of GK rats compared to control sedentary or after exercise. Moderate (8 week) exercise regimen, however, reduced the level of ROS production in the pancreas of GK rats. Similarly, NOX activity was also significantly higher (two to threefold) in GK rat pancreas (Fig. 1B). Exercise training, however, significantly reduced NOX activity in GK rats but not in control rats which remained significantly higher compared to control sedentary rats. On the other hand, pancreatic SOD activity in GK sedentary rats was significantly (~70%) increased and exercise brought the level of enzyme close to the control rats (Fig. 1C). SOD activity in control rat pancreas remained more or less unchanged after 8 weeks of exercise.

**Figure 2:**
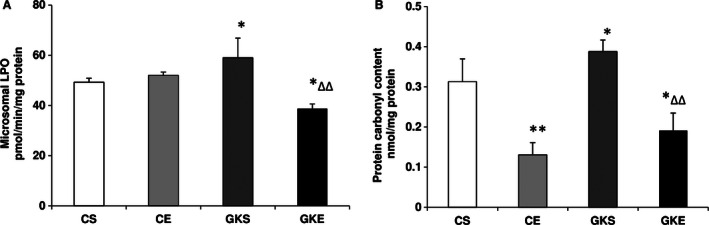
Effect of exercise on Lipid peroxidation (LPO) and protein carbonylation: LPO (A) was measured as thiobarbituric acid reactive substances by measuring MDA formation and protein carbonylation (B) was measured by coupling of oxidatively modified proteins with dinitrophenylhydrazine as described before. The results shown are +/− SEM of three independent experiments. Asterisks indicate significant difference (*P < 0.05; **P < 0.01 from control and ∆ *P* < 0.05; ∆∆ *P* < 0.01 from Goto‐Kakizaki sedentary data).

Lipid peroxidation and peroxidative protein carbonylation were also observed to be significantly higher in the GK rat pancreas (Fig. 2A and B). A marked reduction in the level of lipid and protein peroxidative degradation was observed after exercise. Interestingly protein carbonylation was also inhibited in control rats subjected to exercise regimen. However, there was no significant alteration in LPO in these rats. These results indicate that exercise induces protective mechanisms against ROS‐mediated oxidative stress damages in the pancreas.

**Figure 3:**
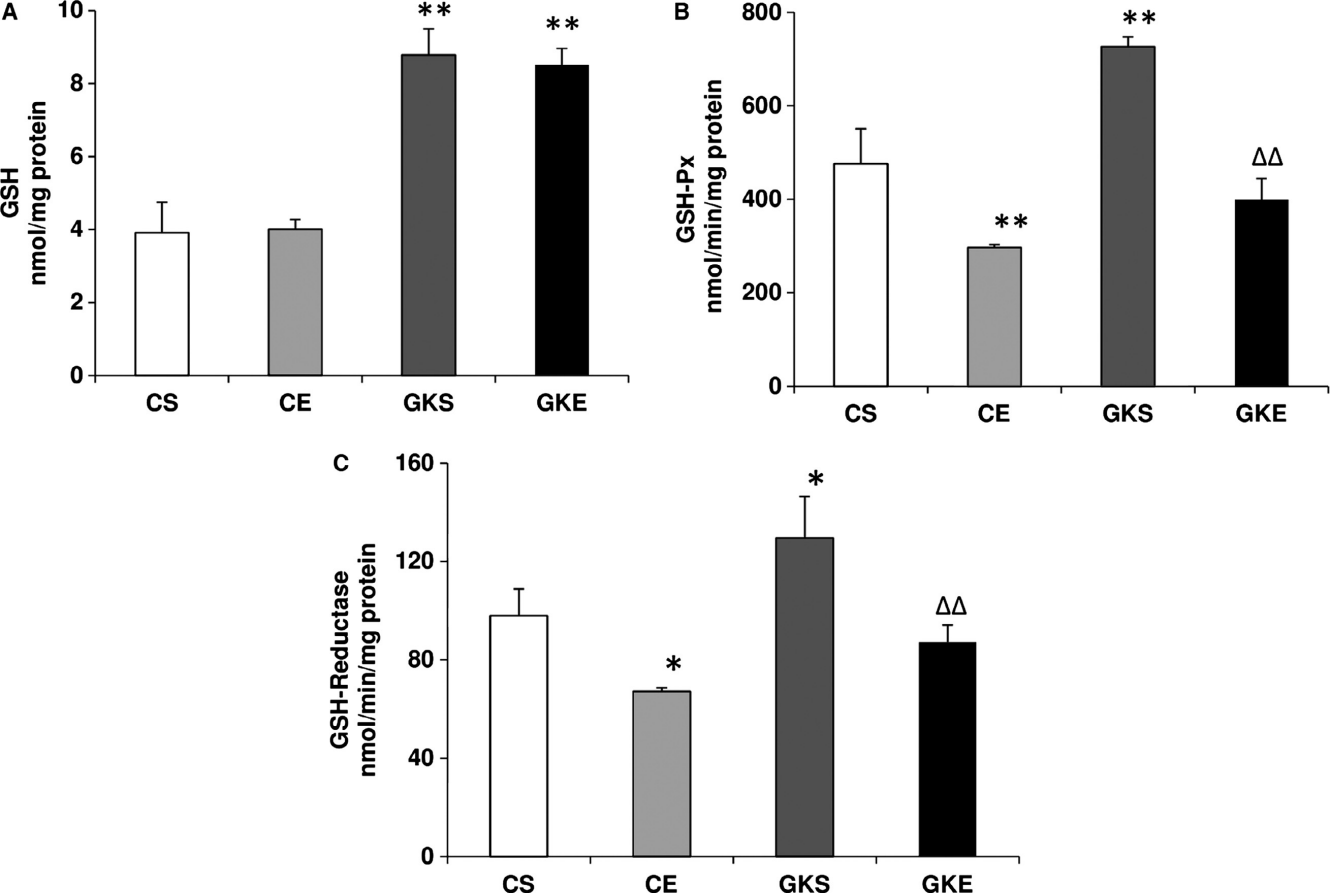
Effect of exercise on GSH metabolism: Reduced GSH (A) was measured in the pancreas from different groups of rats using NADPH‐enzymatic recycling method. GSH‐Px (B) was measured using cumene hydroperoxide as substrate and GSH‐reductase (C) was assayed using oxidized GGSG/NADPH as substrate. The results shown are +/− SEM of three independent experiments. Asterisks indicate significant difference (*P < 0.05; **P < 0.01 from control and ∆∆ *P* < 0.01 from Goto‐Kakizaki sedentary data).

Antioxidant GSH level in the cytosol of pancreas of GK sedentary rats was increased about twofold (Fig. 3A). No appreciable change in the level of GSH was observed even after 8 weeks of exercise regimen suggesting preservation of the increased antioxidant pool in the pancreas of GK rats as a mechanism of cellular defense against the deleterious effects of ROS on pancreatic islets function. GSH‐Px and GSH‐ reductase activities were also significantly higher in sedentary GK compared to control rats (Fig. 3B and C). Exercise training reduced the enzyme activities both in GK and control rat pancreas. This may suggest their significant contribution in preventing and quenching the ROS and oxidative stress in GK rat pancreas.

### Effect of exercise on cytochrome P450 activities

**Figure 4:**
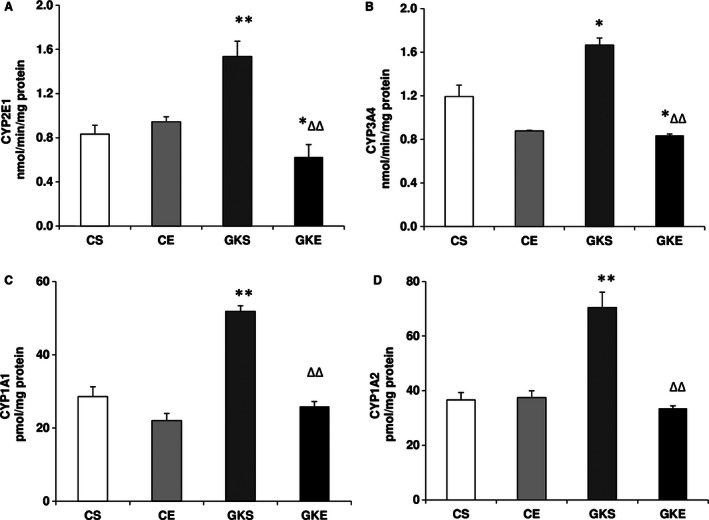
Effect of exercise on cytochrome P450s activities: Microsomal CYPs were assayed using isoenzyme‐specific substrates for CYP 2E1 (A), CYP 3A4 (B), CYP 1A1 (C), and CYP1A2 (D) as described before. The results shown are +/− SEM of three independent experiments. Asterisks indicate significant (<0.05) from control and # indicate significant difference (*P < 0.05; **P < 0.01 from control and ∆∆ *P* < 0.01 from Goto‐Kakizaki sedentary data).

CYP 2E1 plays a significant role in producing ROS and developing oxidative stress in tissues. Figure 4A shows that CYP 2E1 activity increased twofold in the pancreas of GK sedentary rats. 8 weeks of exercise reduced the activity significantly. A similar result was observed with CYP 3A4, CYP1A1, and CYP1A2 activities (Fig. 4B‐D). These results suggest that exercise resulted in the recovery of drugs/ROS metabolizing enzymes altered in diabetes.

### Effect of exercise on energy metabolizing enzymes

**Figure 5:**
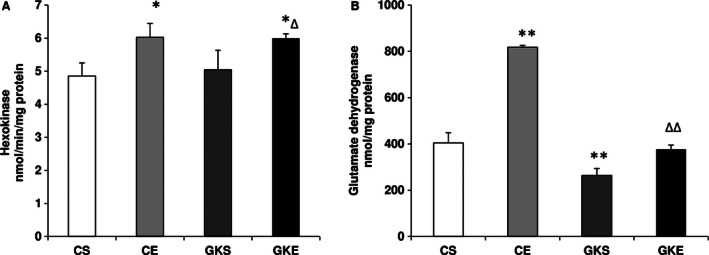
Effect of exercise on hexokinase and glutamate dehydrogenase activities: Hexokinase activity (A) in the pancreas from control and Goto‐Kakizaki (GK) rats on sedentary or after exercise regimen were measured spectrophotometrically as NADH generated using a kit as per the vendor's protocol. Glutamate dehydrogenase activity (B) was measured using a kit as a coupled enzyme assay as per the manufacturer's instructions. The results shown are +/− SEM of three independent experiments. Asterisks indicate significant difference (*P < 0.05; **P < 0.01 from control and ∆∆ *P* < 0.01 from GK sedentary data).

Figure 5A shows that glucose phosphorylation by pancreatic hexokinase enzymes was significantly higher in GK and control rats after 8 weeks of exercise indicating increased metabolism of glucose in the pancreas after exercise. The activity of the mitochondrial enzyme, glutamate dehydrogenase (GDH), on the other hand, was lower in GK sedentary rat pancreas, and increased after exercise (Fig. 5B). These results suggest improved energy utilization in control and diabetic rat pancreas after exercise.

### Effect of exercise on mitochondrial respiratory function

**Figure 6:**
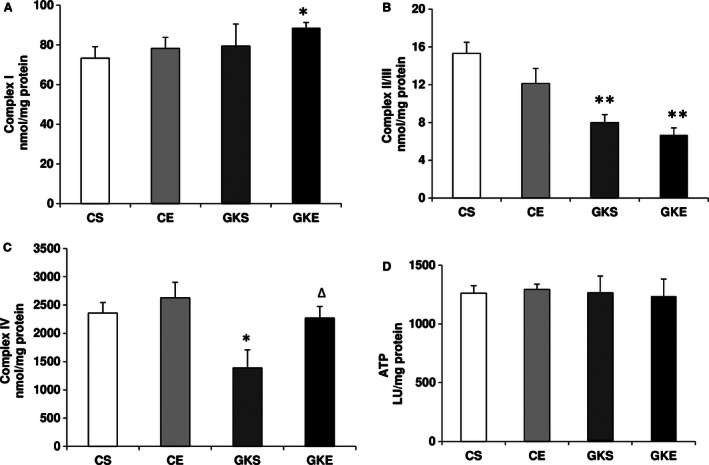
Effect of exercise on mitochondrial respiratory functions: Freshly prepared mitochondrial fractions from control and Goto‐Kakizaki rats on sedentary or after exercise program were assayed using the substrates ubiquinone (A), succinate‐cytochrome c (B), and reduced cytochrome c (C), respectively, by the methods of Birch‐Machin and Turnbull as described in the Materials and Methods. ATP content (D) was also measured by using the bioluminescent assay using a kit from (Sigma, St. Louis, MO) as per the manufacturer's suggestion as described before. The results shown are +/− SEM of three independent experiments. Asterisks indicate significant difference (*P < 0.05; **P < 0.01 from control data).

Figure 6A and C shows a significant increase in the Complex I and Complex IV activities in GK rats after 8 weeks of exercise. While GK sedentary rats exhibited lower Complex IV activity when compared to control sedentary rats, the Complex I activity was not significantly altered. Pancreatic Complex II/III activity (Figure 6B) was, however, significantly (>50%) reduced in GK sedentary rats and exercise made no significant improvement on the recovery of this enzyme. Figure 6D also shows that overall there were no significant alterations in the level of ATP synthesis in the pancreas of sedentary GK rats or after exercise suggesting that the ATP production is preserved to maintain insulin secretion in response to altered glucose metabolism and mitochondrial bioenergetics.

### Effect of exercise on the expression of key redox‐ and energy metabolizing proteins

**Figure 7:**
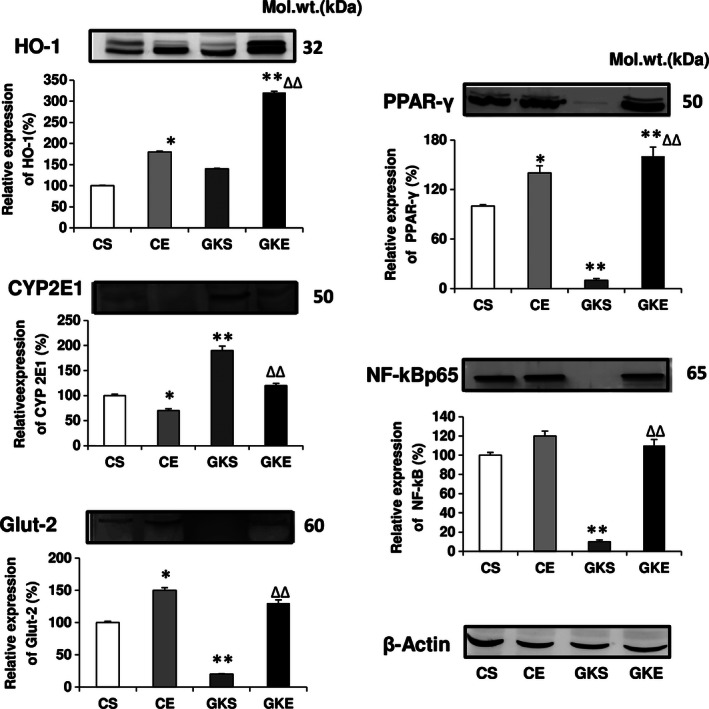
Effect of exercise on the expression of redox proteins: SDS‐PAGE separation of pancreatic proteins using 12% acrylamide gel and transfer on to nitrocellulose paper was performed using standard procedures as described in the Materials and Methods. Expression of HO‐1, NF‐kBp65, CYP2E1, GLUT2, and PPAR‐*γ* was measured by their immunoreactivity with specific antibodies. Beta‐actin was used as loading control. The results shown are representative blots from three independent experiments. Histograms represent the relative expression of the respective proteins (in %age) considering the expression in control as 100%. Molecular weight markers (kDa) are indicated. Asterisks indicate significant difference (*P < 0.05; **P < 0.01 from control and ∆∆ *P* < 0.01 from Goto‐Kakizaki sedentary data).

Figure 7 shows a marginal increase in the expression of redox regulatory protein, heme oxygenase 1 (HO‐1) in the pancreas of GK rats which was significantly increased after 8 weeks of exercise, suggesting the induction of HO‐1 in response to increased oxidative stress. Similarly, CYP 2E1 expression was also increased in GK sedentary rats and was reduced after exercise. NF‐kB p65 protein translocation to nucleus was significantly increased in the pancreas of GK sedentary rats, causing a marked reduction in cytosolic NF‐kB pool. Exercise led to a recovery of the cytosolic NF‐kB expression in GK rats. These results may suggest that the activation of redox‐regulation mechanism in the pancreas might, in part, be associated with NF‐kB signaling.

Another significant observation was the marked increase in the expression of GLUT 2 and PPAR‐*γ* following exercise, which was markedly reduced in GK diabetic rats. These results indicate that both glucose uptake and lipid metabolism have improved in the pancreas of exercising diabetic GK rats.

## Discussion

Insulin resistance in a number of peripheral tissues and an inadequate *β*‐cell response despite normal or even increased amounts of circulating insulin have been implicated in progressive type 2 diabetic complications and metabolic syndrome. Physical exercise has been shown to improve energy metabolism and pancreatic function and may even be considered as more effective than the most widely used antidiabetic drug, metformin (James et al. 1984; Fahien and MacDonald 2011; Kim et al. 2013). The physical exercise effects in diabetes and on insulin resistance involve multiple mechanisms, including insulin‐independent‐enhanced glucose transport, energy metabolism and altered mitochondrial functions, and ROS production (Simoneau and Kelley 1997; Zierath 2002; Powers and Jackson 2008). Prolonged oxidative stress and ROS have a causal role in diabetes which triggers many antioxidative and metabolic adaptive responses (Ristow et al. 2009; Howarth et al. 2011) However, very little is known about the effects of physical exercise on oxidative stress, mitochondrial function and adaptive energy and antioxidant responses in the pancreas of type 2 diabetic rats. We, therefore, have studied the effects of exercise on pancreas using GK diabetic rats subjected to moderate exercise for 8 weeks and compared with control rats subjected to the same exercise training program. Our results have clearly demonstrated increase in oxidative stress in the pancreas of GK rats as observed by the increased production of ROS, LPO, protein carbonylation, and enhanced SOD and NOX activities. While exercise training had no significant effects on the level of total ROS production, SOD enzyme activity or LPO in control rats, it significantly reduced these oxidative‐stress‐associated changes in GK rats suggesting differential mechanisms of ROS production and/or clearance in control and GK rats. Interestingly, NOX, which represents an additional nonmitochondrial respiratory enzyme source of ROS production, was markedly increased in GK sedentary as well as in the control rats subjected to exercise training. In contrast, exercise significantly reduced the elevated NOX enzyme activity in GK diabetic rats, suggesting differential adaptation of ROS production and metabolism in control and GK rats. Similarly, a significant decrease in SOD activity accompanied by a decrease in LPO was observed only in GK rats subjected to exercise training but not in control rats. Exercise training, however, inhibited oxidative carbonylation of proteins both in control as well as in GK rats. These results suggest a differential antioxidative adaptation response in the pancreas of GK and control rats after exercise which may be associated with the different sources of ROS production, and their clearance by antioxidant redox metabolism. There are similar reports (Houstis et al. 2006; Ristow et al. 2009; Guariguata et al. 2014) suggesting the role of ROS metabolizing enzymes, such as SOD and catalase, in diabetes and exercise‐induced adaptive response in peripheral tissues. Improved islet structural integrity and *β*‐cell function in ZDF rats have been reported after exercise training (Rawal et al. 2013). Our results suggest a triggering of antioxidant response in GK rat pancreas after exercise training as evidenced by the increased energy metabolism supported by decreased translocation of NF‐*κ*B to the nucleus, increased expression of Glut‐2 receptors and increased glucose phosphorylation by hexokinase (glucokinase) enzyme activity. This was also confirmed by the increased expression of pancreatic HO‐1, an antioxidant marker, after exercise training. In addition, an improved energy metabolism, as seen by the increased expression of PPAR‐*γ* which regulates fatty acid uptake and metabolism in the pancreas of GK rat was also observed after exercise training. Many studies have reported the use of synthetic ligands of PPAR‐*γ* for treating patients with type 2 diabetes because they restore sensitivity to insulin (Staels and Fruchart 2005). PPAR‐*γ* agonists promote free fatty acid (FFA) uptake and storage in subcutaneous adipose tissue. This reduces FFA levels, thus reducing insulin resistance. In addition, activation of PPAR‐*γ* is believed to increase the expression and translocation to the cell surface of the glucose transporters GLUT‐1 and ‐4, thus increasing glucose uptake into liver and skeletal muscle cells and reducing plasma glucose levels. Our results also indicate an increased glutamate dehydrogenase activity in the pancreas of GK as well as control rats after exercise suggesting improved bioenergetics. Pancreatic *β*‐cell GDH enzyme has been implicated in the regulation of insulin secretion and resistance in peripheral tissues (Giacco and Brownlee 2010). Our results have shown that GDH activity was reduced in the pancreas of GK rats and exercise training increased the activity suggesting an improvement in *β*‐cell function and insulin release along with improved glucose transport and energy metabolism. Our previous studies using type 1 and type 2 diabetic rat models and studies by others have suggested the implication of increased protein carbonylation in oxidative stress conditions in mitochondrial dysfunction and insulin resistance (Raza et al. 2004, 2011, 2012, 2013, 2015; Frohnert and Bernlohr 2013). Our results in this study have also shown a marked inhibition of protein carbonylation and improvement in mitochondrial respiratory function after exercise training which might be implicated in improved insulin sensitivity and energy metabolism. The increased expression of NF‐*κ*B, a redox gene regulating protein and PPAR‐*γ*, responsible for lipid uptake and metabolism in tissues, also improved after exercise, which again suggests improved insulin sensitivity in GK rats after exercise. Increased carbonylation of insulin receptors has been reported to inhibit insulin signaling (Frohnert and Bernlohr 2013). Our results have shown that exercise training inhibited protein carbonylation, both in control and GK rats suggesting an improved insulin signaling. ATP production in the pancreas remained unaffected in GK rats even after exercise training. Complex I activity was not significantly affected in GK rats. However, a significant decrease in Complex II/III and Complex IV activities was observed in GK rats. The inhibition of Complexes II/III and IV in GK diabetic rats may explain the increased ROS production in GK rats by reversing the electron flow backward to produce oxygen‐free radicals (Raza et al. 2004, 2011). Exercise training, however, appears to have improved normal electron transport mechanism and increased oxygen utilization by Complex IV, thereby preventing the leakage of electrons from the respiratory chain. These results may also explain the reduction in ROS levels with improved mitochondrial bioenergetics after exercise in GK rats.

Both cytosolic and mitochondrial energy metabolism by the hexokinase and GDH enzymes, respectively, were found to be increased after exercise training. This may suggest that glucose metabolism is also increased due to increased phosphorylation after exercise. In addition, increase in GDH activity may also improve *α*‐ketoglutarate substrate availability in the Krebs’ cycle, in addition to improving the insulin signaling. Although, we did not measure any specific enzyme involved in the fatty acid oxidation directly, an increased expression of PPAR‐*γ*, suggests an improved uptake and/or metabolism of lipids after exercise training. Further studies are, however, needed to clearly understand whether alterations of pancreatic oxidative stress by exercise are induced by modulation of energy metabolism between glucose and fatty acids. In this regard, we have collected different tissues from control and GK rats subjected to exercise training, and studies are in progress to elucidate the mechanism of insulin sensitivity and improved mitochondrial respiratory bioenergetics. Overall our results suggest that most of the mitochondrial functions in the pancreas improve after exercise training. Our previous studies (Raza et al. 2004, 2011, 2012, 2013, 2015) in diabetic rats have also suggested altered mitochondrial bioenergetics and oxidative stress in the pancreas and peripheral tissues of both type 1 and type 2 diabetic rats.

Our studies have also suggested a significant role of the antioxidant GSH pool and its metabolism in diabetes. Our results have demonstrated that the GSH concentration in the pancreas of GK rats was markedly increased and that this was preserved even after 8 weeks of exercise. This suggests the maintenance of antioxidant GSH pool presumably to prevent the oxidative glutathionylation of proteins in the GK rat pancreas as an adaptation toward increasing oxidative stress by increased ROS production. However, two enzyme systems, GSH‐Px and GSH‐reductase, which are involved in recycling of oxidized GSSG and maintaining the reduced GSH, appeared to be stimulated in the pancreas of GK rats. Exercise training brought their levels to control level. Overexpression of GSH‐Px has been implicated in developing insulin resistance as seen in diabetes and obesity (McClung et al. 2004; Houstis et al. 2006). Our results suggest that exercise may have beneficial effects on insulin sensitivity by inhibiting the GSH‐Px activity in pancreas. Further studies using peripheral tissues are in progress to elucidate the role of exercise on energy and redox metabolism, oxidative stress, and mitochondrial function in GK rats subjected to exercise training.

Our previous studies have also shown alterations in the drug metabolizing enzymes CYP systems in diabetes (Raza et al. 2000, 2004, 2012, 2013, 2015). We have reported that CYP2E1 level in tissues was increased in diabetes (Raza et al. 2004). In this study, we have demonstrated that along with CYP2E1, other CYPs, such as CYP1A1, CYP1A2 as well as CYP 3A4 were increased in the pancreas of GK rats. Exercise training brought their levels close to that of control sedentary rats. No significant change in activities was observed in the control rats after exercise training.

In summary, our results suggest that, in GK rat pancreas, while oxidative stress was increased, the mitochondrial bioenergetics function (such as ATP production) appears to remain more or less steady and exercise has induced adaptive beneficial effects by improving energy metabolism, mitochondrial respiratory function, and inducing antioxidant responses, presumably through NF‐kB signaling. Further studies are in progress to elucidate the role of exercise in peripheral tissues of GK rats after exercise training.

## Conflict of Interest

The authors have no conflict of interest whatsoever.
